# TGF-β contamination of purified recombinant GDF15

**DOI:** 10.1371/journal.pone.0187349

**Published:** 2017-11-21

**Authors:** Oddrun Elise Olsen, Anette Skjærvik, Berit Fladvad Størdal, Anders Sundan, Toril Holien

**Affiliations:** 1 Department of Clinical and Molecular Medicine, NTNU–Norwegian University of Science and Technology, Trondheim, Norway; 2 Clinic of Medicine, St. Olav’s University Hospital, Trondheim, Norway; 3 Department of Biotechnology, NTNU–Norwegian University of Science and Technology, Trondheim, Norway; 4 CEMIR (Centre of Molecular Inflammation Research), Department of Clinical and Molecular Medicine, NTNU–Norwegian University of Science and Technology, Trondheim, Norway; Leiden University Medical Center, NETHERLANDS

## Abstract

Purified recombinant proteins for use in biomedical research are invaluable to investigate protein function. However, purity varies in protein batches made in mammalian expression systems, such as CHO-cells or HEK293-cells. This study points to caution while investigating effects of proteins related to the transforming growth factor (TGF)-β superfamily. TGF-β itself is a very potent cytokine and has effects on cells in the femtomolar range. Thus, even very small amounts of contaminating TGF-β in purified protein batches may influence the experimental results given that receptors for TGF-β are present. When we attempted to characterize possible receptors for the TGF-β superfamily ligand GDF15, striking similarities between GDF15-induced activities and known TGF-β activities were found. However, differences between batches of GDF15 were a concern and finally led us to the conclusion that the measured effects were caused by TGF-β and not by GDF15. Our results emphasize that purified recombinant proteins must be used with caution and warrant proper controls. Notably, some conclusions made about GDF15 in already published papers may not be supported by the results shown. Awareness about this issue in the scientific community may prevent spreading of false positive results.

## Introduction

Growth differentiation factor (GDF)-15, also known as macrophage inhibitory cytokine (MIC)-1, is a distant member of the transforming growth factor (TGF)-β superfamily. Members of the TGF-β superfamily are involved in regulating diverse biological processes, including apoptosis, proliferation, organ development and bone formation. The different ligands are divided into subgroups, including TGF-βs, bone morphogenetic proteins (BMPs), growth differentiation factors (GDFs), activins and inhibins, and nodal.[1] The different ligands signal through type 1 and type 2 receptors that are conserved single transmembrane serine/threonine kinase receptors. The formation of a ligand-receptor complex enables phosphorylation of intracellular SMAD transcription factors. The type of SMAD protein that is activated is determined by the type 1 receptors that are present in the ligand-bound signaling complex. Usually, TGF-β and activins signal through SMAD2/3, whereas BMPs signal through SMAD1/5/8. The activity of TGF-β ligands is also modulated by so-called type 3 receptors, which present the ligand to their type 1 and type 2 receptors.

GDF15 is a stress-activated cytokine that during physiological conditions only is found at high levels in the placenta.[2] Elevated serum levels of GDF15 are found in many pathological conditions such as different types of cancer, metabolic disorders, and cardiovascular disorders like atherosclerosis and coronary heart disease and may be regarded as a common marker of disease and mortality.[3–5] Like TGF-β, GDF15 is proposed to act primarily as an anti-inflammatory molecule. One way GDF15 showed anti-inflammatory properties was by inhibiting leukocyte integrin activation required for survival of mice after myocardial infarction.[6]

GDF15 was reported to induce phosphorylation of SMAD2/3 in cultured neonatal cardiomyocytes in mice,[7] whereas a study using rat cardiomyocytes found that GDF15 induced phosphorylation of SMAD1/5, but not of SMAD2.[8] Other studies have suggested that GDF15 may use TGFBR2 as type 2 receptor by a SMAD- or non-SMAD pathway.[4, 9–12] More recently, TGFBR1 was proposed to be the type 1 receptor for GDF15 by two independent groups.[13, 14]

Myeloma cell lines have well-known responses to many TGF-β superfamily ligands.[15–17] Thus, they represent a possible model system to characterize receptor usage by GDF15, given that GDF15 activates one or both of the SMAD-pathways downstream of TGF-β superfamily type 1 receptors. The initial aim of this study was to show by which receptor(s) GDF15 could signal by use of the myeloma cell lines and other relevant cell types.

## Materials and methods

### Cell culture

The human multiple myeloma cell line INA-6 was a kind gift from Dr. M. Gramatzki (University of Erlangen-Nurnberg, Erlangen, Germany), whereas IH-1 was established in our laboratory.[18, 19] INA-6 cells were grown in 10% heat-inactivated fetal calf serum (FCS) in RPMI-1640 (RPMI) supplemented with recombinant human interleukin (IL)-6 (1 ng/mL), and IH-1 cells were maintained in 10% heat-inactivated human serum (HS) (Department of Immunology and Transfusion Medicine, St. Olav’s University Hospital, Trondheim, Norway) in RPMI and IL-6 (2 ng/mL). The human monocytic cell line THP-1 (ATCC, Rockville, MD, USA) was grown in 10% FCS in RPMI with 50 μM 2-mercaptoethanol. Cells were cultured at 37 ^o^C in a humidified atmosphere containing 5% CO_2_. For experiments 2% HS in RPMI was used, with IL-6 (1 ng/mL) added for INA-6 and IH-1, unless otherwise stated. Experimental medium for THP-1 was 1% FCS in RPMI with 50 μM 2-mercaptoethanol.

### Reagents

Recombinant human GDF15 was mainly Chinese hamster ovary (CHO) cell line-derived (Cat# 957-GD, R&D Systems/Bio-Techne, Abingdon, UK). The two lots of GDF15 that were mostly used here were later tested for TGF-β content by R&D Systems: Lot# EHF1713081 (purchased early 2014) showed 169.7 pg TGF-β per μg GDF15 and Lot# EHF0914051 (purchased late 2014) showed 0.73 pg TGF-β per μg GDF15 and all lots sold since these measurements were done had to pass a quality control of maximum 20 pg TGF-β-content per μg GDF15 (R&D Systems, personal communication). We also performed experiments with “mammalian cell culture”-derived GDF15 (Cat# 120–28, Lot# 1111S396, Peprotech, London, UK) and *E*. *coli*-derived GDF15 (Cat# ab125769, Abcam, Cambridge, UK). Other recombinant human proteins (activin A, BMP9, ALK1-Fc, ALK5-Fc, TGFBR2-Fc, TGFBR2-isotype 2-Fc ACVR2A-Fc, endoglin-Fc, TGFBR3-Fc and M-CSF) were from R&D Systems, except IL-6 (Gibco, Invitrogen, Carlsbad, CA, USA). SB431542 was from Sigma-Aldrich (St Louis, MO, USA). TGFBR2 (Cat# AF-241-NA), pan-TGF-β (Cat# AB-100-NA), and GDF15 (Cat# MAB957) neutralizing antibodies were from R&D Systems. Protein G Sepharose 4 Fast Flow (GE Healthcare, Oslo, Norway) was used in the neutralizing antibodies experiment to pull antibodies out from cell culture media.

### Differentiation of macrophages

Human monocytes were isolated by adherence to plastic from Lymphoprep (Axis‐Shield, Oslo, Norway) separated buffy coats (Department of Immunology and Transfusion Medicine, St. Olav’s University Hospital) and grown in 10% human serum in RPMI with M-CSF (15 ng/mL) for ten days before they were used in experiments.

### Western blotting

Cells were treated as indicated, washed with ice-cold phosphate-buffered saline (PBS) and lysed for 30 minutes on ice. The lysis buffer contained 1% IGEPAL CA-630 (Sigma-Aldrich), 150 mM NaCl, 50 mM Tris-HCl (pH 7.5), protease inhibitor cocktail (Roche, Basel, Switzerland), 1 mM Na_3_VO_4_ and 50 mM NaF. Samples were separated on NuPAGE Bis-Tris gels with MOPS running buffer (Invitrogen). Gels were blotted onto nitrocellulose membranes, blocked with 5% nonfat dry milk in Tris-buffered saline with 0.01% Tween 20 (TBS-T) and incubated with indicated primary antibodies. Primary antibodies used were: phospho-SMAD1/5/9 (RRID: AB_2493181, Cat# 13820S), ERK1/2 (RRID: AB_390779, Cat# 4695), SMAD2/3 (RRID: AB_10698742, Cat# 3102S), all from Cell Signaling Technology, Beverly, MA, USA, phospho-SMAD2 (RRID: AB_1587251, Cat# 04–953, Millipore A/S, Oslo, Norway) and GAPDH (RRID: AB_2107448, Cat# Ab8245, Abcam). Blots were washed in TBS-T before incubation for one hour with horseradish peroxidase conjugated secondary antibodies (Dako Cytomation, Glostrup, Denmark). The blots were washed thoroughly with TBS-T before bands were detected using SuperSignal West Femto (Thermo Fisher Scientific, Waltham, MA, USA) as luminescence substrate and Licor Odyssey FC (LI-COR Biosciences, NE, USA).

### QRT-PCR

Total RNA was isolated using the High Pure RNA Isolation Kit (Roche Applied Science, Mannheim, Germany), and complementary DNA (cDNA) was synthesized using the High Capacity RNA-to-cDNA kit (Applied Biosystems, Foster City, CA, USA). PCR was performed using StepOne real-time PCR System and Taqman Gene Expression Assays (Applied Biosystems). The Taqman assays used were: *ACVR1B/ALK4* (Hs00244715_m1), *ACVR1C/ALK7* (Hs00899854_m1), *TGFBR1/ALK5* (Hs00610320_m1), and *GAPDH* (Hs99999905_m1). The comparative Ct method was used to estimate relative changes in receptor expression using *GAPDH* as housekeeping gene.

### Transfections

INA-6 cells were transfected by electroporation using the Nucleofector device (Amaxa biosystems, Cologne, Germany) and Amaxa Nucleofector Kit R (Lonza, Basel, Switzerland) as previously described. Cells were then used for immunoblotting or QRT-PCR 48 hours after transfection. For each transfection cells were treated with either 1 μM ON-TARGETplus Non-Targeting pool, SMARTpool *ACVR1B*, *ACVR1C* or *TGFBR1* siRNAs (Dharmacon RNAi technologies by Thermo Scientific, Lafayette, CO, USA).

### Bio-Plex analysis

TGF-β levels in protein batches of GDF15 were analyzed with Bio-Plex Pro TGF-β1 immunoassay according to the manufacturer’s instructions using a Bio-Plex 200 system and Bio-Plex Pro Wash station (Bio-Rad Laboratories, Hercules, CA, USA). The experiment was performed once.

### Statistical analysis

GraphPad Prism 7 (GraphPad Software, La Jolla, CA, USA) was used to calculate statistical significance. A one-way ANOVA test was used and P-values ≤0.05 were considered statistically significant. Asterisks above bars indicate the degree of significance (*, P≤0.05; **, P≤0.01; and ***, P≤0.001).

## Results

**Fig 1:**
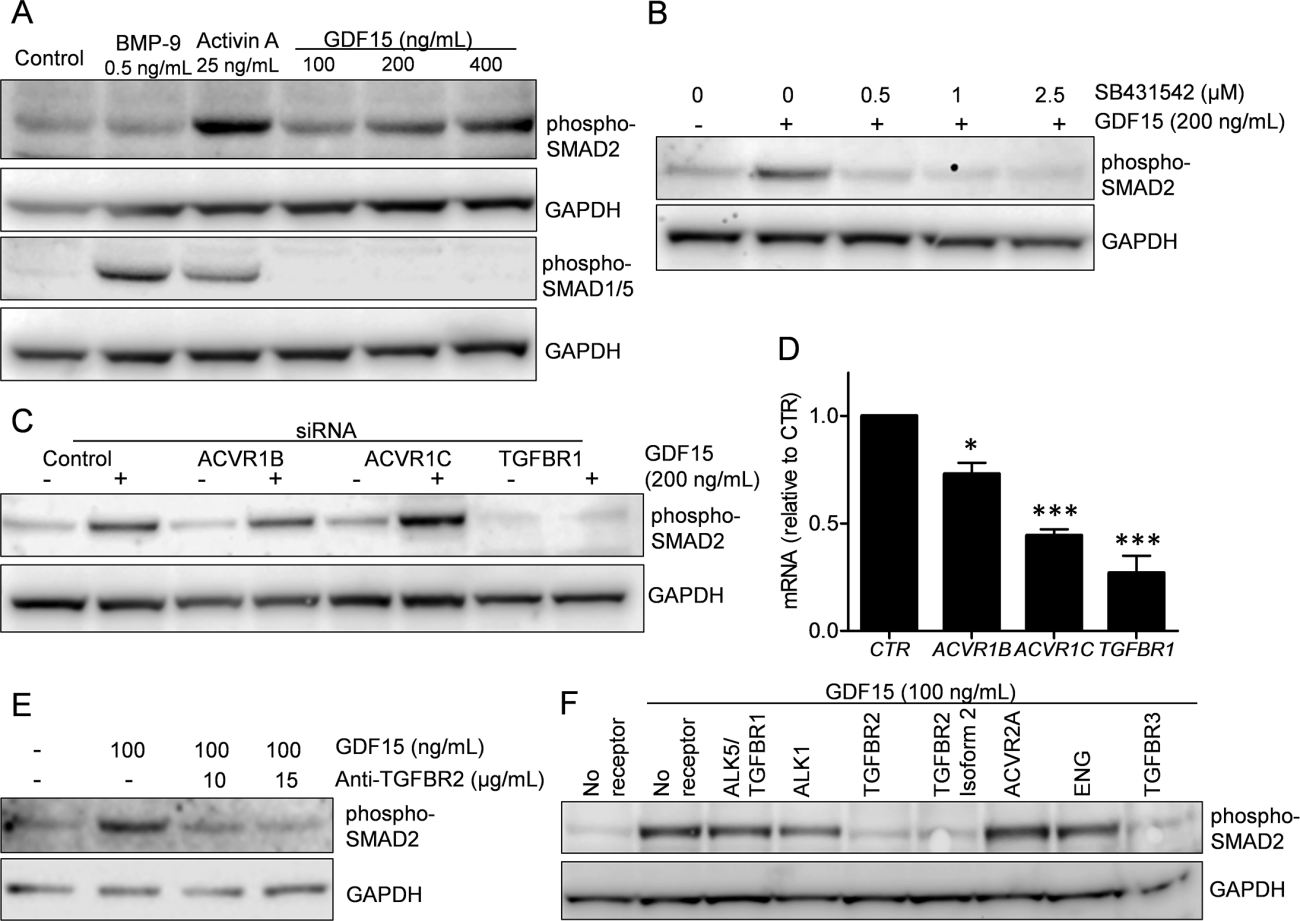
SMAD-activation by recombinant GDF15 in myeloma cell lines. A. Phosphorylation of SMAD1/5 or SMAD2 was determined using immunoblotting in IH-1 cells treated with BMP-9 (0.5 ng/mL), activin A (25 ng/mL) or indicated concentrations of GDF15 (100–400 ng/mL) for 1 hour. B. INA-6 cells were treated with GDF15 (200 ng/mL) and the inhibitor SB431542 (0–2.5 μM) for 1 hour before immunoblotting with anti-phospho-SMAD2. C. INA-6 cells were transiently transfected with siRNAs targeting *ACVR1B/ALK4*, *ACVR1C/ALK7*, *TGFBR1/ALK5* and a non-targeting control siRNA. Two days after transfection the cells were treated with GDF15 (200 ng/mL) for 1 hour before immunoblotting with anti-phospho-SMAD2. D. Knock-down of receptors by siRNA in cells used in (C) as shown by QRT-PCR. Gene expression was calculated with the comparative ΔCt-method with *GAPDH* as housekeeping gene. The error bars indicate SEM of three independent experiments. Asterisks above bars indicate the degree of significance for downregulation of each gene compared to control (*, P≤0.05; **, P≤0.01; and ***, P≤0.001). E. INA-6 cells were treated with GDF15 (100 ng/mL) and a neutralizing TGFBR2 antibody (10–15 μM) for 1 hour before immunoblotting with anti-phospho-SMAD2. F. INA-6 cells were treated with GDF15 (100 ng/mL) and the indicated soluble receptors (5 μg/mL for all except endoglin, which was 1 μg/mL) for 1 hour before immunoblotting with anti-phospho-SMAD2. Antibody staining towards GAPDH was used as loading control for all Western blots. The experiments were performed 2–3 times each. GDF15 used in this figure was from R&D Systems, Lot# EHF1713081.

Based on previous reports on possible involvement of SMAD-activation and use of TGF-β receptors, we wanted to determine if any of the SMAD pathways were activated in myeloma cell lines that express such receptors. We found that recombinant human GDF15 activated SMAD2, but not SMAD1/5 in the multiple myeloma cell line IH-1 (Fig 1A). BMP9 and activin A were used as positive controls for SMAD1/5- or SMAD2-activation, respectively. Interestingly, activin A also activated the SMAD1/5-pathway, and we found this to be actual activin A signaling via a BMP type 1 receptor (Olsen *et al*., manuscript in preparation). SB431542, is an inhibitor of ALK4, ALK5 and ALK7, the TGF-β family type 1 receptors that preferentially activate SMAD2 and/or SMAD3.[20] We have previously shown that SB431542 inhibited activin A- and TGF-β-induced SMAD2 phosphorylation also in myeloma cells,[17] and here we found that GDF15-induced activation of SMAD2 in the INA-6 myeloma cell line was inhibited by SB431542 (Fig 1B). To determine which of the type 1 receptors were involved in GDF15-induced activation of SMAD2, we transiently knocked down *ACVR1B/ALK4*, *TGFBR1/ALK5* or *ACVR1C/ALK7* in the INA-6 cell line using siRNA. Knockdown of *TGFBR1* completely counteracted GDF15-induced activation of SMAD2 (Fig 1C and 1D). Similar results were seen by *TGFBR1* knockdown in human mesenchymal stem cells (S1 Fig). To look for type two receptor usage, we treated INA-6 cells with GDF15 and a neutralizing TGFBR2 antibody (Fig 1E). The antibody inhibited GDF15-induced activation of SMAD2. Then we looked for possible effects on GDF15-induced signaling by adding soluble chimeric Fc-receptors. Soluble TGFBR2, TGFBR2 isoform 2 and TGFBR3 inhibited GDF15-induced SMAD2-activation, whereas there was no effect of adding soluble TGFBR1/ALK5, ALK1, ACVR2A or endoglin (Fig 1F). In summary, our results indicated that recombinant GDF15 activated SMAD2 in myeloma cells, possibly through the same receptors as TGF-β.

**Fig 2:**
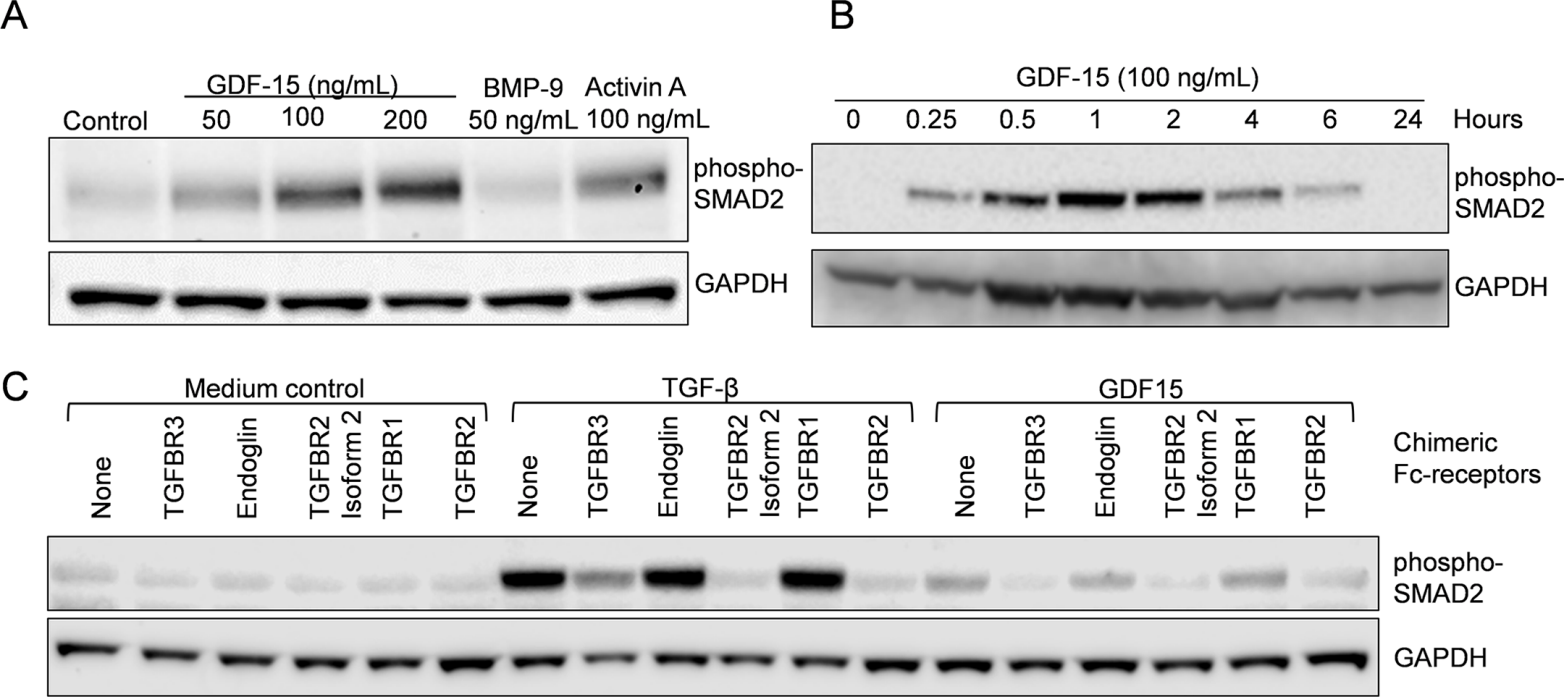
SMAD-activation by recombinant GDF15 in THP-1-cells and *in vitro* differentiated macrophages. A. Monocytic THP-1 cells were treated with GDF15 (50, 100 or 200 ng/mL), BMP-9 (50 ng/mL) or activin A (100 ng/mL) for 4 hours. B. THP-1 cells were treated with GDF15 (100 ng/mL) for various time-points. C. *In vitro* differentiated macrophages were treated with indicated soluble receptors in the presence of TGF-β (1 ng/mL) or GDF15 (200 ng/mL) for four hours. Phosphorylation of SMAD2 was determined using immunoblotting and GAPDH was used as loading control for all Western blots. Each experiment was performed once. GDF15 used in this figure was from R&D Systems, Lot# EHF1713081.

We then wanted to see if GDF15 also activated SMAD2 in other cell types and performed experiments on the THP-1 monocytic cell line and *in vitro* differentiated macrophages. Addition of GDF15 dose-dependently (Fig 2A) and time-dependently (Fig 2B) activated SMAD2 in THP-1 cells in a manner like in the myeloma cell lines. We then wanted to compare receptor-binding between TGF-β and GDF15. Thus, monocytes (adherent PBMCs) were differentiated into macrophages *in vitro* before addition of TGF-β, GDF15 and different chimeric Fc-receptors. Both TGF-β and GDF15 caused phosphorylation of SMAD2, but TGF-β was much more potent than GDF15 (Fig 2C). More interestingly, the pattern of inhibition of TGF-β- or GDF15-induced SMAD2-activation seen by addition of chimeric Fc-receptors was identical.

**Fig 3:**
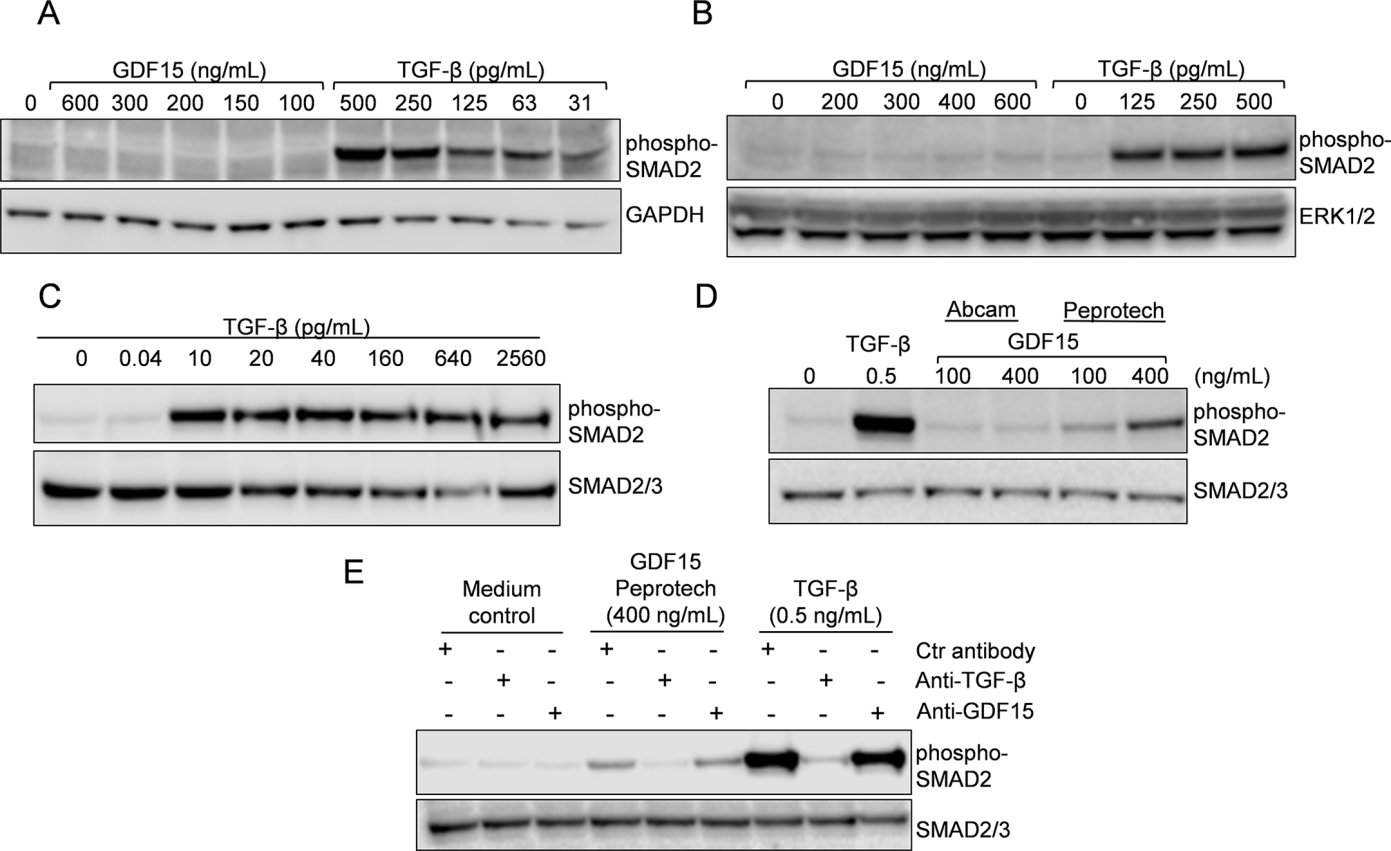
Activation of SMAD2 by recombinant GDF15 was caused by TGF-β. *In vitro* differentiated macrophages (A) or THP-1 cells (B) were treated with increasing doses of recombinant GDF15 (R&D Systems, Lot# EHF0914051) or TGF-β for four hours. C. INA-6 cells were treated with increasing doses of TGF-β for 1 hour. D. INA-6 cells were treated for 1 hour with the indicated doses of TGF-β, GDF15 (Abcam) or GDF15 (Peprotech). E. INA-6 cells were treated for 1 hour with GDF15 (Peprotech) or TGF-β pre-treated with neutralizing antibodies targeting GDF15 or TGF-β. For C-E, the experiments were performed in RPMI with 0.1% bovine serum albumin (BSA). Phosphorylation of SMAD2 was determined using immunoblotting and GAPDH, ERK1/2 or SMAD2/3 antibodies were used as loading controls. All experiments were performed at least three times, except for D and E, which were performed twice.

Due to the striking similarities between TGF-β and what we thought was GDF15, we aimed to do more control experiments. However, our earliest batch of GDF15 (169.7 pg TGF-β per μg GDF15, see Materials and methods section) was empty and the newer, purer batch of GDF15 (0.73 pg TGF-β per μg GDF15) did not cause phosphorylation of SMAD2 in macrophages, THP-1 cells (Fig 3A and 3B) or INA-6 cells (S2 Fig). Nonetheless, TGF-β potently induced SMAD2 phosphorylation, even at doses down to 10 pg/mL (~ 420 femtomolar) (Fig 3A–3C). A TGF-β dose of 0.04 pg/mL, which equals the amount of TGF-β that would have been present in the purer batch if GDF15 had been used at 50 ng/mL, did not activate SMAD2 (Fig 3C). All the cell types used expressed potential SMAD2-activating receptors, thus the lack of effect was not due to lack of receptors (S3 Fig). We then looked for other sources of GDF15 to be able to control for the observed effects. A batch of mammalian expressed GDF15 obtained from Peprotech induced SMAD2 activation, whereas a batch of *E*. *coli*-expressed GDF15 from Abcam did not (Fig 3D). Although we cannot tell if the *E*. *coli*-expressed GDF15 was properly folded and biologically active, the combined results support a possible TGF-β contamination of some batches of recombinant GDF15 derived from mammalian cell culture. To show if the SMAD2 activity induced by recombinant GDF15 from Peprotech was caused by TGF-β or by GDF15, we incubated the recombinant proteins with neutralizing antibodies and pulled them out with protein G sepharose before treatment of cells. A neutralizing antibody targeting GDF15 had no effect, whereas a TGF-β specific antibody completely abrogated the activation of TGF-β as well as GDF15 (Fig 3E). Furthermore, using a TGF-β1 Bio-Plex assay we measured the TGF-β content in the GDF15 batches from Peprotech, R&D Systems (Lot# EHF1713081), and Abcam to be 82, 27, and 0 pg per μg GDF15, respectively. Thus, we conclude that at least in the cells tested here: myeloma cell lines, human mesenchymal stem cells, the monocytic THP-1 cell line and monocyte-derived human macrophages, we found no evidence that pure GDF15 was able to activate SMAD2.

## Discussion

Researchers should be aware that the purity of preparations of recombinant proteins varies, and thus, our results may not be surprising. Nevertheless, by searching Pubmed it is hard to find proper literature describing this issue, especially if you are not already aware of the problem. The use of very high doses of recombinant proteins increases the probability of encountering problems with highly potent contaminants. There are, however, many publications that indicate or support both that GDF15 should be used in high doses to obtain biologically relevant effects and that GDF15 might signal through TGF-β receptors. The decision to use relatively high levels of GDF15 for this study was based on previously published results and the levels proposed by different suppliers. Typically, the activity of GDF15 in cellular assays has been reported at concentrations around 1–3 μg/mL by suppliers. Specifically, Peprotech stated that the activity of their mammalian expressed GDF15 was “determined by its ability to inhibit alkaline phosphatase activity in differentiating MC3T3/E1 osteoblast cells. The expected ED50 for this effect is 1.0–3.0 μg/mL”. Likewise, Abcam stated that the activity of their *E*. *coli*-expressed GDF15 was “determined by inhibition of DU-145 cells and is typically 1–2 (g/mL)”. These concentrations are even higher than the ones used by us (50–400 ng/mL). Notably, even if suppliers use a limit of 20 pg TGF-β per μg GDF15, this may not be sufficient to avoid unwanted effects on cells since TGF-β works in the femtomolar range as shown here and by others.[21]

The CHO cell line constitutively secretes TGF-β that is functional in human cells.[22] It has also been shown that TGF-β specifically bound to nickel and was co-purified with histidine-tagged proteins during immobilized metal affinity chromatography (IMAC).[23] Contamination of functional TGF-β has been found in purified human chorionic gonadotropin (hCG) preparations from JEG-3 cells.[24] Furthermore, functional TGF-β has been found to contaminate bovine bone matrix-derived beta 2-microglobulin and preparations of recombinant murine Wnt3a derived from mammalian cell culture.[25, 26]

Our study emphasizes the need for protein preparations of high purity. Purified recombinant proteins must be used with caution and be accompanied by proper controls. More alarmingly, unwanted effects by contaminating proteins may not have been discovered in already published papers. Published results based on purified recombinant proteins must thus be interpreted with care. Interestingly, three independent groups recently found GDNF family receptor α-like (GFRAL) to be the receptor for GDF15, supporting our findings.[27–29]

## Supporting information

S1 Fig SMAD2 activation by recombinant GDF15 through TGFBR1/ALK5 in human mesenchymal stem cells. Human primary mesenchymal stem cells were transfected with Non-targeting, *ACVR1B/*ALK4 or *TGFBR1/*ALK5 siRNA and treated with GDF15 (200 ng/mL) for 1 hour. Phosphorylation of SMAD2 was determined using immunoblotting and GAPDH was used as loading control. The experiment was performed once. GDF15 used in this figure was from R&D Systems, Lot# EHF1713081.
(TIF)

S2 Fig Effect of recombinant GDF15 and TGF-β on SMAD2 activation in INA-6 cells. INA-6 cells were treated with increasing doses of recombinant GDF15 (R&D Systems, Lot# EHF0914051) or TGF-β for 1 hour and subjected to Western blotting with antibodies targeting phospho-SMAD2 or ERK1/2 as a loading control. The figure shows one of two independent experiments.
(TIF)

S3 Fig Relative expression levels of potential SMAD2 activating receptors. Expression of TGF-β superfamily receptors *ACVR1B*/ALK4, *ACVR1C*/ALK7, *TGFBR1*/ALK5, *TGFBR2*, *TGFBR3*/betaglycan, and *ENG*/endoglin was determined in IH-1, INA-6, THP-1 and in vitro differentiated macrophages using QRT-PCR. The delta delta Ct method using *GAPDH* as housekeeping gene was used to determine the relative levels of mRNA compared to the expression of *ACVR1C* in macrophages (Ct-value = 36) was set to 1. The values are representative for one out of three independent experiments. The error bars represent 1 SD of technical triplicates.
(TIF)

S1 File Supporting materials and methods. (PDF)

S1 Table Data points for Fig 1D. (XLSX)

S2 Table Data points for S3 Fig. (XLSX)
