# Supplementary figures and images for: TGF-β contamination of purified recombinant GDF15
Source: PLoS One. 2017 Nov 21;12(11):e0187349. doi: 10.1371/journal.pone.0187349 (PMC5697882; doi:10.1371/journal.pone.0187349)

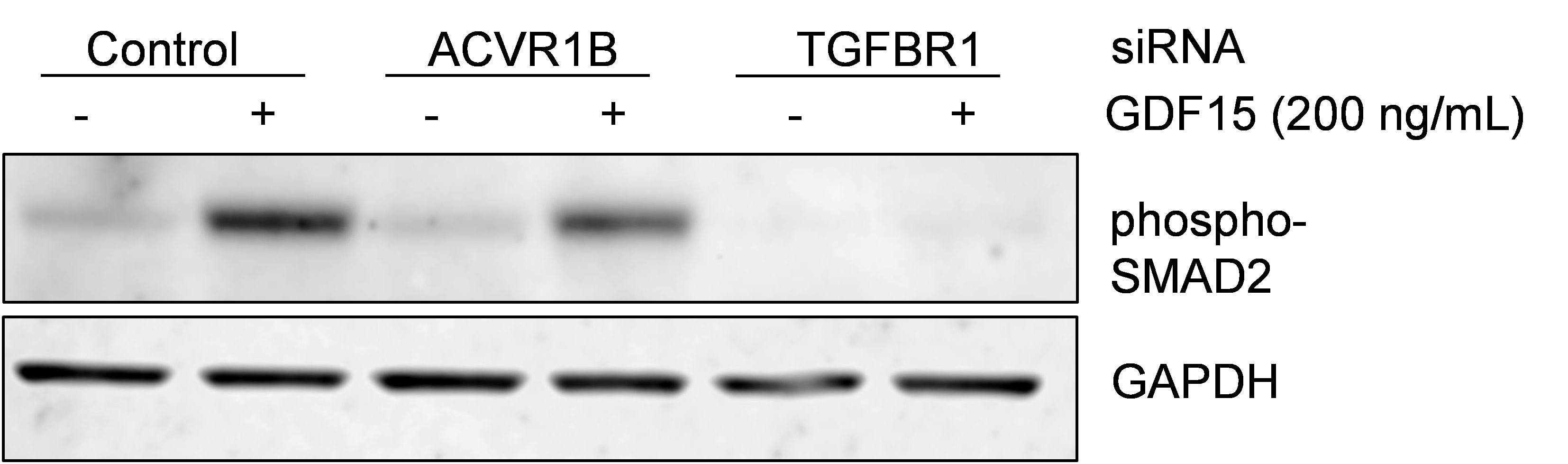

Supplement: S1 Fig — Human primary mesenchymal stem cells were transfected with Non-targeting, ACVR1B/ALK4 or TGFBR1/ALK5 siRNA and treated with GDF15 (200 ng/mL) for 1 hour. Phosphorylation of SMAD2 was determined using immunoblotting and GAPDH was used as loading control. The experiment was performed once. GDF15 used in this figure was from R&D Systems, Lot# EHF1713081. (TIF) [file pone.0187349.s001.tif]

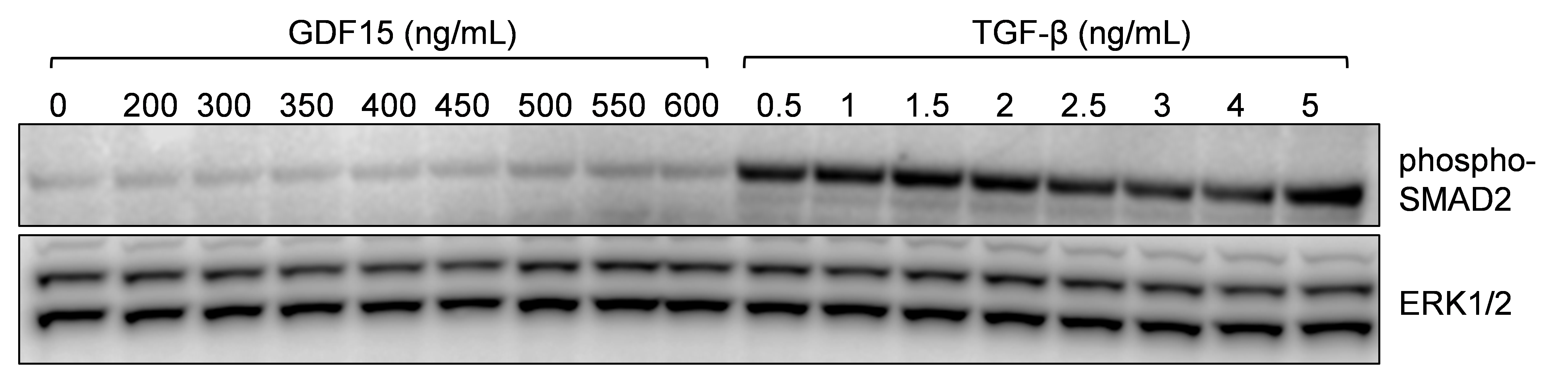

Supplement: S2 Fig — INA-6 cells were treated with increasing doses of recombinant GDF15 (R&D Systems, Lot# EHF0914051) or TGF-β for 1 hour and subjected to Western blotting with antibodies targeting phospho-SMAD2 or ERK1/2 as a loading control. The figure shows one of two independent experiments. (TIF) [file pone.0187349.s002.tif]

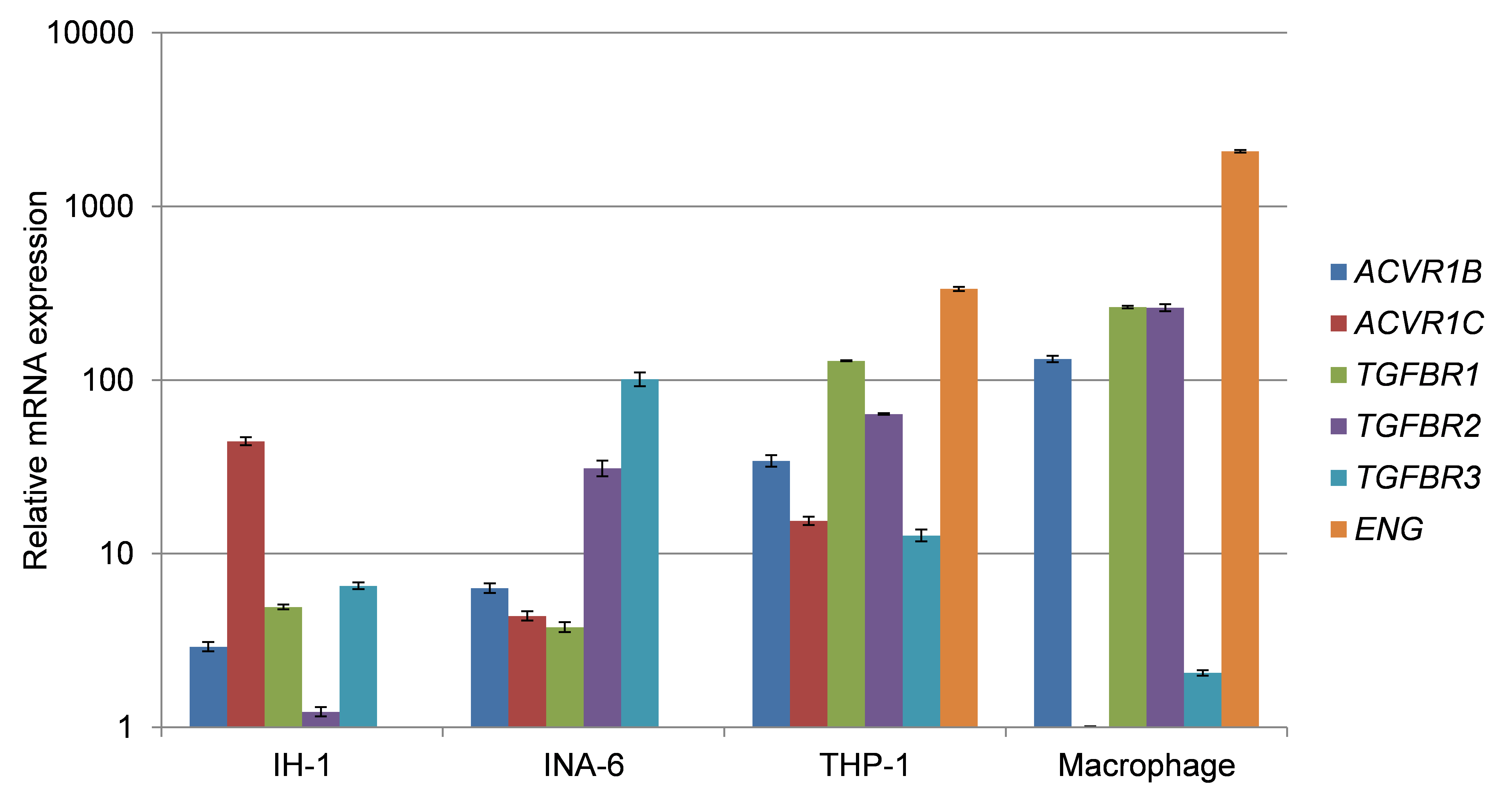

Supplement: S3 Fig — Expression of TGF-β superfamily receptors ACVR1B/ALK4, ACVR1C/ALK7, TGFBR1/ALK5, TGFBR2, TGFBR3/betaglycan, and ENG/endoglin was determined in IH-1, INA-6, THP-1 and in vitro differentiated macrophages using QRT-PCR. The delta delta Ct method using GAPDH as housekeeping gene was used to determine the relative levels of mRNA compared to the expression of ACVR1C in macrophages (Ct-value = 36) was set to 1. The values are representative for one out of three independent experiments. The error bars represent 1 SD of technical triplicates. (TIF) [file pone.0187349.s003.tif]
