# Supplementary material for: TGF-β contamination of purified recombinant GDF15
Source: PLoS One. 2017 Nov 21;12(11):e0187349. doi: 10.1371/journal.pone.0187349 (PMC5697882; doi:10.1371/journal.pone.0187349)
Supplement: S1 File — (PDF) [file pone.0187349.s004.pdf]

# S1 File

# Supporting materials and methods

## Transfection of human mesenchymal stem cells

Mesenchymal stem cells (MSC) (Lonza, Walkersville, MD, USA) were cultured in mesenchymal stem cell growth media (Lonza) at 37 °C in a humidified atmosphere containing 5 % $CO_2$, according to the manufacturer's instructions. Cells were plated 24 hours before they were transfected with ON-TARGETplus Non-Targeting pool, SMARTpool *ACVR1B*, or *TGFBR1* siRNAs (Dharmacon RNAi technologies by Thermo Scientific, Lafayette, CO, USA) using Lipofectamine RNAiMAX. Two days after transfection the cells were used for experiments.

## QRT-PCR

The QRT-PCR for receptor expression in cells was performed as described in the main manuscript. The additional QRT-PCR Taqman assays used in S3 Fig were: *TGFBR2* (Hs00234253_m1), *TGFBR3* (betaglycan) (Hs01114253_m1), and *ENG* (endoglin) (Hs00923997_g1).
